# On the Performance of Generative Adversarial Network by Limiting Mode Collapse for Malware Detection Systems

**DOI:** 10.3390/s22010264

**Published:** 2021-12-30

**Authors:** Acklyn Murray, Danda B. Rawat

**Affiliations:** Department of Electrical Engineering and Computer Science, Howard University, Washington, DC 20059, USA; db.rawat@ieee.org or

**Keywords:** generative adversarial networks, algorithms, mode collapse, long short-term memory, generator model, discriminator, machine learning, malware, intrusion detection system

## Abstract

Generative adversarial network (GAN) has been regarded as a promising solution to many machine learning problems, and it comprises of a generator and discriminator, determining patterns and anomalies in the input data. However, GANs have several common failure modes. Typically, a mode collapse occurs when a GAN fails to fit the set optimizations and leads to several instabilities in the generative model, diminishing the capability to generate new content regardless of the dataset. In this paper, we study conditional limiter solutions for mode collapse for the Intrusion Detection System (IDS) Control Flow GAN (ICF-GAN) model. Specifically, the ICF-GAN’s mode collapse instances are limited by a mini-batch method that significantly improves the model accuracy. Performance evaluation is conducted using numerical results obtained from experiments.

## 1. Introduction

Generative adversarial networks (GAN) are unsupervised learning models, which comprise of a generator and discriminator, determining patterns and anomalies in the input data [1]. The GAN conditionally produces outputs that have the probability of being drawn from the original dataset [2]. In GAN, mode collapse occurs when a GAN fails to fit the set optimizations and leads to a number of instabilities in the generative model that diminishes the capability to generate new content regardless of the dataset [3]. Generally, there are four reasons for mode collapse in GAN [3]. First, when the objective of GAN is misleading, the singular generator generates output predictively, but the model’s discriminator evaluates the output based on authenticity instead of outputs diversity. In that situation, the generator would generate a huge number of examples while the capacity of the discriminator is comparatively low. In this way, the discriminator cannot direct the generator to approximate distribution. Second, when ignoring the objective function, the generator accounts for individual examples without considering backward or forward samples. In this case, it does not incentivize the generator correctly, which results in an irregular output. Third, the problem of mode collapse can be explained in terms of gradient exploding where there is imbalance or inconsistency exists between generator and discriminator. Four, when the discriminator process is inefficient and processes each example one by one (in an independent manner) and the mechanism of distinguishing the output fails. In short, the actual problem in this whole process is based on mode discovery and mode retention. The idea is related to generator optimization; allowing the recognition of possible data clusters balancing the focus to fool the discriminator.

**Figure 1:**
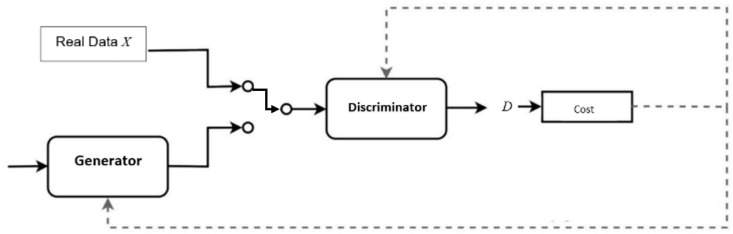
Generative adversarial network.

In this paper, we proposed to enhance the GAN-enabled ingestion process for PCAP-based malware detection (i.e., Intrusion Detection System) similar to the proactive analysis Causal Analysis based on System Theory (CAST) model [4,5]. The Network-based IDS (NIDS) control flow model is a hybrid system engineering monitoring method for cybersecurity and safety hazards in industrial automated networked environments and cyber tabletop disaster mitigation [6,7]. We leverage the mini-batch methodology to limit the mode collapse instances with improved accuracy. We leverage the PCAP for malware detection while limiting the mode collapse to ensure diversity in the proposed IDS Control Flow GAN (ICF-GAN) ingestion process. Figure 1 illustrates a typical GAN with input data, generator, discriminator, etc.

The proposed ICF-GAN model abridges the layered process for flow-based network performance rather than adopting a distance association for data distribution for the benefit of system model ingestion with limited overfitting as the goal. The organization of this paper includes the related works influencing the proposed method, the framework of the generative adversarial network with the problem statement, dataset comparison for the proposed method, and performance metrics of the proposed ICF-GAN.

## 2. Related Work

Image synthesis is a grave issue in the domain of computer vision, where GANs showed substantial advancements [2]. High detailed features can be traced by the discriminator in far-off fractions of the image that are consistent with each other. The proposed solution added to the convolutions with an intention to achieve long-range modeling and multiple level dependencies [2]. Synchronization with a distant portion makes the model capable of drawing fine details of the image. The stabilization of the model was attained by training models for the difficult datasets that were considered challenging and helpful in analysis for a solution to our problem statement. Spectral normalization and two times scale update rule were used to effectively tweak generator and discriminator while addressing the problem of slow learning of discriminators [2]. Zhang et al. compared the developed two models by adding in distinct layers of the network while training these models for one million iterations. They observed inception scores and Frechet inception distance [2]. Evaluation of residual blocks with equal parameters as the self-attention blocks showed that self-attention blocks performed better.

Ghosh et al. worked in multi-agent diverse generative adversarial networks considering the problem of mode collapse [8]. The architecture of the model is based on the minimax game where they employed the modules of the generator and discriminator [8]. The discriminator in the study performs the usual task of classification of images in real or fake groups while the generator maximizes the error rate of the discriminator [8]. During this process, the model is trained to produce images that are close to real images. Moreover, the problem of mode collapse persists that they targeted. The proposed solution is to enhance the usability of multiple generators by coupling the sum with only one discriminator, which is a feature in our research. The authors allowed participatory generators to exchange information based on the initial layer parameters of the generator [8]. The purpose of information sharing is the limitation of the initial layer to capture low-frequency structures with the goal of reduction in redundant computations [8]. In case of exception, where an image with entirely distinct modalities exists, it is recommended to discontinue the parameter sharing. To prevent the generator from generating similar samples, they modified the objective of discriminator as well as its process of categorizing the image as real or fake. They made their discriminator learn to push generated outcomes from all generators towards diverse identifiable modes. Enforcement of diversity allowed generating plausible images.

Schmidhuber presented unsupervised neural networks in context with game theory and minimax-based games [9,10]. Schmidhuber discussed the adversarial curiosity with two types of networks. First, a network that learns to generate outputs in a probabilistic manner, while the second network makes predictions about the effects of the outputs. The process is dependent on the network that works on maximizing the objective function, which is already maximized (iterative process) [9]. The author further discussed the applications of GAN with the idea of generating adversarial curiosity from GANs. By reviewing the literature, they proved that predictability minimization is not dependent or based on minimax games.

Furthermore, alternative methods address mode collapse issues with varieties of proposed GAN architectures, including Wasserstein GAN (WGAN) [11], unrolled GAN [12,13], AdaGAN [14], VEEGAN [15], zero-entered gradient penalty on training examples (GAN-0GP) [16], zero-entered gradient penalty on interpolated samples (GAN-0GP) [17] and nudged-Adam (NuGAN) [18]. These architectures have been computed on different distributions of data as well as both synthetic and real datasets. The architectures address different metrics methods for indicating mode collapse in a model and conventionally solve the problem of mode collapse.

## 3. GAN Framework and Problem Statement

The intention of our research is to provide an RNN layer in a Generative Adversarial Network to optimize incrementally rebuff mode collapse instances in the ICF-GAN model.

### 3.1. Brief Overview of GAN and Loss Functions in GANs

In a GAN, as shown in Figure 1, a generator and a discriminator are associated with an independent loss function. The generator and discriminator train completely unsupervised by the process and output sample sets due to the loss functions [19]. The dual loss functions accommodate the sought-after unsupervised learning capabilities within the GAN, but the functions produce costs in training [20]. The discriminator cost is that of identification of real samples as real and the correct identification of generated samples as generated with *L* as the loss function.

(1) $$L^{\left(D\right)}\left(\theta^{\left(D\right)}\;,\;\theta^{\left(G\right)}\right)=-\frac{1}{2}E_{xp_{data}}\log D\left(x\right)-\frac{1}{2}E_{z}\log\left(1-D\left(G\left(z\right)\right)\right)$$

where *D* represents a discriminator and *G* denotes a generator for batch training), *x* represents a real sample, *z* represents the random noise vector, *G*(*z*) denotes the data generated by the generator, and E represents the expectation. *D*(*x*) indicates the probability that *D* discriminates *x* as real data, and *D*(*G*(*z*)) indicates the probability that *D* determines the data generated by *G*. The goal of *D* is to correctly determine the source of the data, *D*(*G*(*z*)) to approach 0, while the goal of G is to increment to 1. Due to the duality of interest, there exists a conflict between these two models (i.e., zero-sum game). Therefore, the loss of the generator derives from the discriminator. On the left, where the first term identifies real samples as real, and the right term identifies generated samples as generated [19]. The generator cost is the negative of $\tau_{D}$ representing the discriminator that is,

(2) $$\tau_{G}=-\tau_{D}$$

With this variation of the loss function, the generator depicts strong signals when the function outputs samples that are discriminator-generated identifications [21]. By utilizing (2), a variation of a two-player minimax game formulation is developed with the training performing as forecast and increased stability as represented for a dataset in (3).

(3) (V(D,G)G        D  minmax= G        D  minmax(ExPdata(x)[logD(x)]+EzPz(z)[log(1−D((z))])

### 3.2. Limiting Mode Collapse

The tackling of mode collapse is intuitively counter-positive to a one-pattern procedure due to mode collapse variations on applications. Although a series of methods can be of impactful importance in using GAN on multimodal data, the use of different methods, independently modified or combined, can effectively archive the issue.

- *Class Grouping*: class grouping is a non-complex, tedious yet effective method for novices to tackle modal collapse. This is the pattern of identifying the verities and classes in the dataset [22]. After the identification process, parsing into groups is conducted according to the goal feature similarities carrying different classes. The method ensures fewer mode opportunities to bypass the discriminator, empowering the discriminate against sub batches and identifying a fake or real batch.
- *Counter Actions Anticipation Pattern*: in this method, the actions taken can be said to be a vice versa of grouping from an outlook because the main action focuses more on removing the situation of the discriminator and halting by analyzing and embracing the generator’s output to effectively outsmart the discriminator [23]. This is conducted by using the discriminator’s pattern and using vetted patterns as counter-action against collapse. This method has worked effectively in different operations, but also it is limited due to the increase in time as a result of training and number of increased gradient calculations [23].
- *Using Past Experince And Data*: in reference to the name, using past data generated by the generator to pre-produce fake samples, prior to creating the fake samples with the data set, the discriminator is then trained with those samples fixed number of iterations [12].
- *Implementing Multiple Networks*: the chosen foundational method for our study, or different modal class, assists in holistically covering the classes of the network data. The generated data quality, as well as increased time to train, are noted disadvantages.

The key limiting feature of our research occurs prior to the generator failure to capture modes during training. Alternate methods have been proposed to address the problem of mode collapsing in GANs. Most of these methods claimed to address the mode collapsing problem. However, most of these methods lack comparison for their performance. Due to the data set and identification method enacted, the options available in tackling mode collapse for our GAN scenario include objective functions, architecture modification, and mini-batch discrimination [12]. 

### 3.3. Alternate Collapse Limiter Methods

Alternative methods address model collapse issues with varieties of proposed GAN architectures, including Wasserstein GAN (WGAN) [11], unrolled GAN [12,13], AdaGAN [14], VEEGAN [15], zero-entered gradient penalty on training examples (GAN-0GP) [16], zero-entered gradient penalty on interpolated samples (GAN-0GP) [17] and nudged-Adam (NuGAN) [18]. These architectures were computed on different distributions of data as well as both synthetic and realistic or real datasets. The architectures address different metrics methods for indicating model collapse in a model and conventionally solve the problem of mode collapse.

Wasserstein GAN (WGAN) was proposed based on the observation that the Jensen–Shannon distance in the standard GAN architecture was not adequate as the cost function used to learn the distributions of low dimensional manifolds [11]. Therefore, the authors of WGAN proposed an alternative cost function called the Wasserstein distance, which posed a more natural measurement of the distance between two probability distributions [11]. The Wasserstein distance computes the amount of effort needed to move from one distribution to another and has been regarded as a sensible function when comparing two probability distributions. However, it is difficult to compute it in practice. To mitigate this challenge, the authors also proposed the calculation of just the approximate Wasserstein distance. This was achieved through clipping the weights that ensure that the learned function was k-Lipschitz and not the supremum over all 1-Lipschitz. The proposed method was not specifically designed to address mode collapse; however, the authors claimed that the new Wasserstein distance facilitated stabilization during the training process and, therefore, avoided mode collapse [11].

The VEEGAN was proposed as an additional reconstruction network that was used to invert the generator network, thus mapping the generated samples to some random noise and thus exploring variational principles to estimate implicit probability distributions in avoiding mode collapse [15]. To achieve this, regularization was introduced to penalize the difference between the reconstruction function and the generator function [15]. It is stated that the regularization method cannot be computed easily, therefore, Jensen’s inequality is used [15]. Both the generator and reconstruction network were trained together with the introduction of the implicit variation principle. To achieve better performance, the author prescribed pre-training the reconstructor before the entire training process [15].

By using the machine learning boosting technique, AdaGAN learns a mixture of GANs [14]. The authors achieved this by learning the weights of the network over the training set according to some heuristics. After the weights were computed, training of a component GAN on a reweighed training set was achieved at some step [14]. The component GAN model was then added to the current mixture model according to an established scheme. By doing this, the authors proved that the developed method reduces the *f*-divergence that exists between the distribution represented by the mixture model (reweighed version of the true data distribution) and the true data distribution [13]. The reweighing is a function of the mixture weight and the density ratio of the mixture model distribution, and the true data distribution [14]. The density ratio is computed from a function of the mixture discriminator network trained to separate points from the mixture model and the training set [13]. Therefore, the component GAN is learned through the training on its corresponding reweighed training set.

The UnrolledGAN employed unrolled optimization for the discriminator network to develop a surrogate objective for the generator update [11]. The generator, therefore, has an opportunity to unroll *n* steps and establish how the discriminator can optimize itself [11]. After this, the generator is updated through the backpropagation algorithm, and the cost function is computed in the last step. This prevents the generator from exploiting local optimal that can be easily distinguished by the discriminator, thus lowering the chance that the generator will be overfitted for a specific discriminator, and, therefore, reducing mode collapse and eventually improving stability [11].

The authors in [24] evaluated and compared the performance of WGAN, AdaGAN, VEEGAN, and Unrolled GAN concerning mode collapse limitations. The experiments were performed over both synthetic and real datasets using the same architectures and training procedures for the GAN algorithms. All the GAN methods were compared across five different metrics on the dataset. The 2D distribution was easily learned by the networks, therefore, all the GANs generated samples provided Wasserstein distance that is close to the test samples. For the coverage metrics (also measures the distance between real and generated sample distribution), VEEGAN recorded the highest coverage on the 2D ring dataset, WGAN and AdaGAN recorded the highest coverage on the 2D ring dataset. The WGAN generated low quality samples [24].

For the high dimensional synthetic dataset with nine modes, AdaGAN was able to capture all the modes while VEEGAN was able to generate samples that were closest to the real data, had the highest quality samples, and captured six modes. As for Unrolled GAN, it failed to capture any modes [24]. The experiment also covered MNIST real dataset. The experiment showed that AdaGAn had the lowest Wassertein distance while all methods except VEEGAN were able to achieve a score of 0.9 for coverage metrics and captured each of the 10 modes [24]. The authors of [24] also applied second-order gradient information to tackle the issue of mode collapse. They analyzed the loss surface from its Hessian eigenvalues and thereby showed that mode collapse directly relates to convergence towards sharp minima. The work observed how the eigenvalues of the generator network were directly correlated to the occurrence of mode collapse and designed an optimized algorithm nudged Adam (NuGAN. It overcomes mode collapse by using spectral information, which leads to a stable convergence property.

Several other methods were also proposed recently to either mitigate the issue of mode collapse. Such methods included SD2GAN [25], IID-GAN [26], VirtualGAN [27], Pluggable Diversity Penalty Module [28], SSGAN [29], MGGAN [30], and MGO-GAN [31]. 

Siamese Dual Discriminator Generative Adversarial Network (SD2GAN) [25] authors proposed a Siamese Dual Discriminator network to reduce the impact of mode collapse and some other limitations. The proposed network consists of a Siamese Network and an additional discriminator within a regular GAN network. The idea is to encourage the generator network to learn all available modes from realistic images in the training set and generate more realistic and diverse samples.

The proposed network is evaluated against other methods such as WGAN and MGGAN using Inception Score (IS) [32], Frechet Inception Distance [33], and Coverage Metric evaluation metrics. The inception score is used to evaluate both the quality and the diversity of the data generated by the network. Frechet Inception Distance (FID) metric is used to measure the similarity with the real data and the generated data by computing the Gaussian distribution from the features, mean, and covariance [33].

IID-GAN [26] employed regularization with an independent and identically distributed (IID) sampling perspective to show that it can work to avoid mode collapse. The authors also proposed a new loss function to effectively compute the distance between the real distribution and the target distribution.

The authors of VirtualGAN [27] proposed a framework that mitigates the effect of mode collapse in GANs. They employ the concept of virtual mapping in the training of the GAN by integrating two processes that merge and split into the GAN network. Multiple data points are merged into one before the discriminator is trained in the merge process, which allows the generator network to capture merged-data distribution [27]. The split process is applied after training is completed to split the output of the generator, thereby producing diverse modes, therefore, reducing the mode collapse problem.

The proposed Pluggable Diversity Penalty Module (PDPM) [28] intends to eliminate mode collapse in GANs by forcing the generator network to generate samples with distinct features if they possess different latent vectors. The feature map of fake samples is extracted from the discriminator network first. Afterward, a normalized Gram matrix is employed to determine the similarity between the feature maps [28]. PDPM is used to penalize the generator if two latent vectors that have low similarity are mapped to fake samples with similar features. This process thereby reduces the occurrence of mode collapse.

GANs with supervision signal SSGAN [29] uses supervision signal to inform the generator of the approximate output that corresponds to the input noise and ensures that the generated distribution is similar to the real distribution, therefore, making sure that the generator can capture the data distribution better and potentially eliminating mode collapse. The authors also proposed a new evaluation metric called matching score. 

MGGAN [30] proposed the manifold-guided GAN algorithm to tackle mode collapse. It employs a guidance network that already exists on the GAN architecture to induce the generator network to learn all the modes that exist in a data distribution. The guidance network effectively represents the overall modes in a data distribution, which helps penalize mode imbalance [31]. Mitigates mode collapse by employing multiple generator GANs that are based on orthogonal vectors, which are used to calculate the difference between two feature vectors to show the correlation between them [31]. The orthogonal value is minimized through back-propagation during training and integrated into the generator loss to update the training parameters, therefore, helping to eliminate mode collapse during training.

The architecture methods, such as the multi-agent diverse GAN (MAD-GAN), deal with mode collapse by incorporating several generators and a single discriminator [34]. The multiple generators are tasked to capture diverse samples. Secondly, the MAD-GAN is designed to identify the fake and real samples and find the generator that generated the counterfeit samples. 

The other architecture technique used in limiting mode collapsing in the generative adversarial network is by mode regularized GAN (MRGAN) [35]. MRGAN argues that mode collapse is caused by failure to penalize the generator for the missing modes. Mode regularized GAN uses an encoder that tries to match real data and generated manifolds. 

Unrolled GAN solves the mode collapse problem and stylizes the GAN training [15]. In the unrolled GAN, the generator is allowed to predict discriminator response. This prediction is made possible by using surrogate objective functions for the generator [36]. The generator unrolls the discriminator k steps for the current discriminator update [37]. The standard generative adversarial network differs from the unrolled generative adversarial network as the generator’s update in unrolled GAN is performed based on the *k* step update of the discriminator given the current generator update [38].

Mode collapse being the major concern faced by GAN, the research focused on determining the best method to limit the mode collapse and make the GAN more stable [39,40,41,42,43,44,45]. When tackling a particular problem, integrated LSTM networks focus on merging the best characteristics of several components. Adding capacity and depth to an LSTM network is as simple as stacking the LSTM layers. Multilayer completely linked structure is another way to think about it. Researchers have embraced the stacked LSTM network because of its simple and effective construction. Vehicle-to-vehicle communication was studied using the stacked LSTM network by Du et al., who discovered that the stacked LSTM-based regression model performed far better than logistic regression [46]. The task of translating from English to French was completed using a stacked LSTM network with four layers and 1000 cells per layer. This LSTM network’s performance was enhanced when the source words were reversed, as this introduced short-term dependencies between the source and the target sentence. Because of this, the LSTM with several layers performed better than one with only a few layers [47]. Yao et al. highlighted the fact that because the erroneous signals from the top must be backpropagated via multiple layers of nonlinear transformations, they may be lessened or exploded in the stacked LSTM network [48]. 

Note that in LSTM, it is not uncomplicated to process long sequences effectively by padding and truncating strategies, which deals with opcode sequence [49]. A fixed length of N is first selected. If the length exceeds, the excess length is truncated to be equal to N. When it is less than N, padding to make its length as N, which is injected as a predefined identifier. Such a strategy ensures that the process remains constrained in length and consistent. The challenge of this strategy is that most of the sequence information is not used [49].

Our goal is to study to minimize mode collapse in a GAN framework purposed for malware detection based on PCAP data where data ingestion to the discriminator in GAN impacts the data modifications, control modifications or adaptations for the model. We leverage a single-layer long short-term memory (LSTM) for mini-batch integration for better detection and overall accuracy. 

## 4. Proposed Approach

We proposed an alternate method that uses mini-batch discrimination optimization to solve GAN mode collapse. Mini-batch discrimination is used in addressing mode collapse problems in the GAN in different contexts for image processing, which is different from our applications. In the mini-batch-based discrimination in GAN, the discriminator looks at mini-batches of samples but not individual samples to limit the generator’s mode collapse [12,32]. In this concept, as the discriminator considers a batch of samples, it could be easier to spot a single-mode collapse. The discriminator understands when all the samples (data) in the batch are closer to each other, the data could be fake [32]. The generator, therefore, could be forced to generate many outputs that are good from each batch of samples [32]. Work in [12] studied mode collapse limiters for over-optimization of the generator and used a generator loss function that incorporates the current discriminator’s classifications, whereas the work in [32] considered the vanishing gradient inclusions for the discriminator of a GAN for image-based applications. Similarly, PCAP data overfitting reduction was studied in [50] for generator data balancing, whereas, in our proposed approach ICF-GAN, the LSTM unconditionally ingested into the discriminator for system model injection for integration and PCAP data overfitting reduction by leveraging LSTM layer optimization without overfitting by labeling and loss function modification. 

A version of mini-batching gradient descent compares a loss function, L(w), of the PCAP model parameters, randomly sampled in a set $B_{t}$, in each iteration. Our learning rate is exemplified in $\propto_{t}$ and the estimate by the stochastic gradient presents as $\nabla_{w}L_{B_{t}}\left(w_{t}\right):$

(4) $$w_{t}-\propto_{t}\nabla_{w}L_{B_{t}}\left(w_{t}\right)=w_{t}+1$$

Further, the outputs of $V_{w}L(w_{t})$ from the mini-batch values are an unbiased estimator to assist in the data balancing and limiting overfitting.

(5) $$var\;\left(V_{w}L_{B_{t}}\left(w_{t}\right)\right)\propto \frac{n^{2}}{b}\;var(\nabla_{w}\;L\left(w_{t)}\right)$$

**Figure 2:**
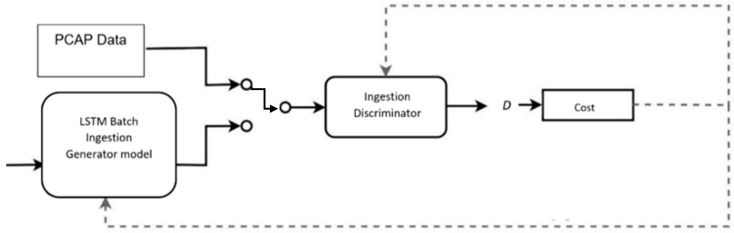
ICF-GAN Ingestion Model with LSTM.

Our proposed method offers mitigation of mode collapse by limited mini-batching for training speed and accuracy for malware detection while appending the similarity in the discriminator with a single dense LSTM layer in the discriminator, as shown in Figure 2.

**Figure 3:**
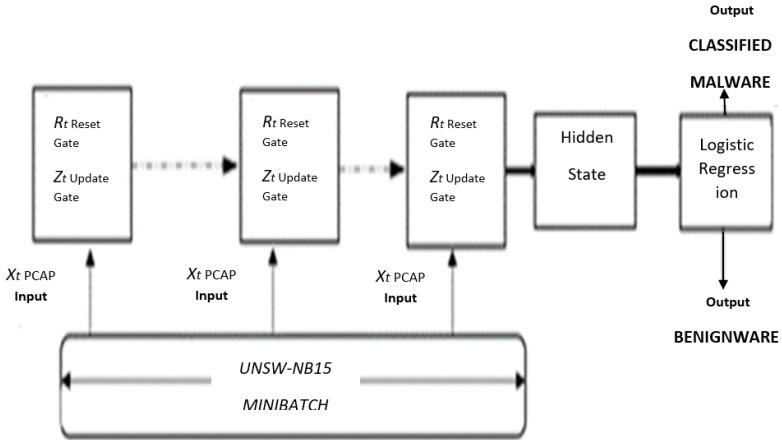
Proposed mini-batch hybrid architecture using LSTM.

Due to the proposed architecture, a series of LSTM for the mini-batch methodology, mode collapse is presented. LSTM, long short-term memory, is a variant of recurrent neural networks (RNNs) and the cornerstone in deep learning for sequential predictions. The proposed model has a series of LSTM similar to a Gated Recurrent Unit (GRU), and the LSTM with a combination of in an updated state from the forget and input gates is leveraged [44]. A merger of the cell state and hidden state provision the mini-batch predictions as output. After limiting mode collapse accounts are modeled, performance can be more improved through additional training. Our goal is to enhance the accuracy of malware detection with a mode collapse limiter. Figure 3 below shows the LSTM architecture.

With proper architectures that can deal with the challenges when developing malware detection methods, it is possible to protect our device or software from malicious malware. As explained above, the LSTM network processes and predicts based on time series data for malware detection. LSTM can be used directly to ingest raw opcode sequences, which are extracted decompiled files limiting such instances. Note, a series of opcode sequences lengths are problematic for the LSTM to train because of the gradient vanishing problem [49]. Using a better hierarchical structure on opcode sequences is possible to learn long opcode sequences for the hybrid LSTM model presented bespoke for PCAP file ingestion. 

Using a better hierarchical structure on opcode sequences is possible to learn long opcode sequences for the Hybrid LSTM model presented bespoke for PCAP file ingestion. Compared to alternate PCAP mini-batch models, PacketCGAN by P. Wang et al. elicited CGAN constraint condition parameters for the generator data balancing. In ICF-GAN, the LSTM is unconditionally ingested into the discriminator for system model injection for integration and PCAP data overfitting reduction based on limited training [50]. Similarly, a counterpart PCAP model, with the goal of accuracy, applied the Earth Moving distance method to a logistic regression detection model can be impacted by overfitting [51]. The logistic regression detection model was trained on the same data set as ICF-GAN [51,52]. Unlike Wasserstein distance methodology nor the feature extraction, by duel conditional frequency and spatial parsing presented in Deep-IRTarget, the ICF-GAN proposed model addresses the gap in PCAP discriminator, limited LSTM layer optimization without overfitting by labeling and loss function modification, prior to ingestion in a Control Flow model with capacity limitations for detection sensitive alignment [53,54,55,56,57]. 

## 5. Datasets

In developing the GAN-based malware detection, misapplication and anomaly-based approaches were evaluated. IDS uses a misuse-based strategy to proportion analysis to align the characteristics of previously established network attacks. The tool archive is maintained regularly by storing established network attack patterns. On the other hand, anomaly-based IDS compares unexplained network attacks to normal connection patterns to spot them. Anomaly-based IDSs are thought to be resilient, but they are prone to producing many false positives. The information below analyzes two major datasets utilized in networking: the UNSW-NB15 and the IDS 2017 [41]. The hardware consisted of a Workstation with a 10 core (Intel Core i9-10900X), 256 GB system memory, 1 TB of OS SDD and NVMe3.70 GHz duel RTX 3080 Ti with 12 GB VRAM on an Ubuntu OS. 

### 5.1. UNSW-NB15 Analysis

Raw network packets were produced using the IXIA Perfect Storm method to produce the UNSW-NB 15 dataset in the Australian Centre for Cyber Security’s Cyber Range Lab. The method provides a combination of true contemporary normal tasks and artificial current attack behaviors inclusive of 100 GB of raw traffic in the form of PCAP archives by the Tcpdump method [52]. To produce 49 features with the class mark, the Argus and Bro-IDS tools were used, creating 12 algorithms. They created a suitable dataset for testing a network anomaly detection system.

The characteristics of the UNSW-NB15 dataset were divided into six categories: flow features that entail characteristics that distinguish hosts, such as client-to-server or server-to-client [40]. The attributes that reflect protocol relations are included in these functions [52]. Content characteristics whose attributes include TCP/IP attributes, as well as certain HTTP service attributes. It also has time features whose category includes time-related attributes such as packet delivery time, start/end packet time, and TCP protocol round trip time. Secondary generated features are a category that can be further bisected into two categories: general-purpose features and protocol-specific features [40]. The flow of 100 record connections originating from the last time function’s sequential order is used to create link functions. Finally, the Labelled Feature is a group that reflects each record’s name.

Machine learning algorithms that use clustering and outlier identification do not necessitate database upgrades. Many researchers have focused on machine learning-based IDS for misuse anomaly and hybrid identification using the UNSW NB-15 dataset [52]. Finding methods for detecting sophisticated cyber-attack requires a thorough examination of various machine learning approaches [40]. Single classifiers using all data set features, multiple classifiers using all features of the data set, single classifiers using restricted features of the data set, and multiple classifiers using limited features of the dataset are used in machine learning-based IDSs.

### 5.2. The Intrusion Detection Evaluation Dataset (IDS-2017)

The most important security tools against advanced and ever-growing network threats are IDSs and intrusion prevention systems (IPSs). Anomaly-based intrusion detection methods suffer from stable and precise performance evolutions due to a lack of adequate test and confirmation datasets. Since 1998, researchers have evaluated 11 databases and found that most were out of date and inaccurate [40]. The series of overviewed analyzed datasets lack traffic diversity and scale. Although a pinnated number of datasets cover the full range of documented threats, anonymize packet payload info, categories the sets as unfit to represent current trends.

### 5.3. CICIDS2017 Analysis

This dataset includes innocuous and modern malware attacks (circa 2017) and nearing resemblance of grounded network results (PCAPs). It also provides the effects of a network traffic review using CICFlowMeter, which includes labeled flows depending on the source, timestamp and destination IP addresses, destination ports, protocols, and attack vectors (interred in CSV files). The description of the extracted features is available for in-depth analysis. The researcher’s top priority in creating this dataset was to generate accurate context traffic [41]. The majority appear to have used the B-Profile method to profile abstract human activity behavior and generate naturalistic neutral context traffic. Experts used HTTP, HTTPS, SSH, FTP, SMTP, POP3, and IMAP protocols to create the abstract behavior of 25 users for this dataset.

### 5.4. Multi-Agent Diverse Malware Model

Our algorithm supports the collapse limiter measures for malware identification in the UNSW-NB 15 dataset [52]. The IDS Control Flow ingestion method delineates the predictable identification signatures from the PCAP data, with the reiterative GAN improving the analysis by introducing the adoption of novel data packets. This approach reviews the system risk factors by Control Flow constraints, which allows for better comparison and evaluation.

**Figure 4:**
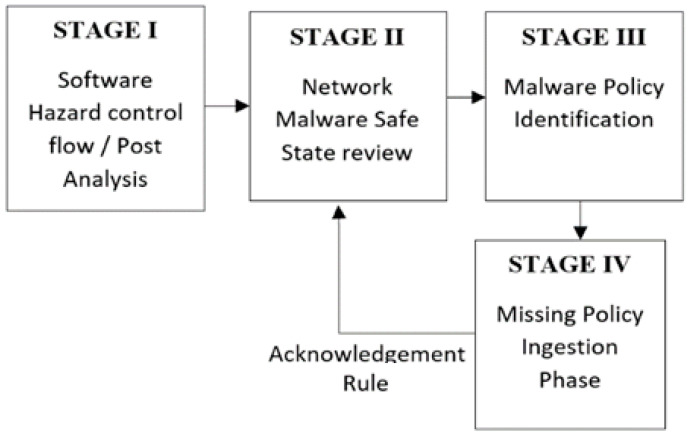
Re-ingestion Flow Diagram.

Our hybrid IDS Control Flow method associates malware traces, by mapping malicious data with benign data, to either existing in-network machines or external to the current network [1]. STAGE I primes the ingestion for software hazards control flow issues, in Figure 4. STAGE II-tier networked malware identification compares safe state activities. STAGE III is the malware detection phase, and STAGE VI acts as a missing policy information ingestion stage for GAN model optimization.

Acknowledgment rules for the control flow re-ingestion are novel identification policies cycled and ingested from the GAN model. The malware identification phase with STAGE VI aligns the missing control flow information agent within the IDS Control Flow model with malware Control Flow rules of identifying malware by creating policies that compare certain signature characteristics. The avoidance of overfitting, a goal of the proposed ICF-GAN model, is enhanced by the layered LSTM and output integrated into the ingestion process.

Emanating from System Operations and Control Structure hazards, the Acknowledgement re-ingestion parses the categorization based on protocol, known state signatures, by packet capture identifications. The LSTM mini-batch layers accommodate identifications strings by the Generator for post-injection. The GAN Discriminator ingests the network data for analysis of the recursive algorithm for the IDS (see Algorithm 1).
**Algorithm 1.** LSTM Mini-batch LayerX represents the number of cells
$S_{j}$ represents the # of PCAP cell
$\mathrm{Input}\;x=[x_{1}$$,\;\ldots ..x_{128}$$],\;x_{i}$
Given parameters:

Initialize
For t=1, …, 128 do$\;\theta_{j}=\theta_{j}-\alpha \frac{1}{128}\;\sum_{k=i}^{i+8}(h_{\theta }\left(x^{\left(k\right)}\right)-y^{\left(k\right)})x_{j}^{\left(k\right)}$(for every j=0,…., 128)
End for
for j=1 to X$\mathrm{Output}:\;\Delta w_{out_{j}m}$$=\alpha \delta_{out_{j}y_{x^{m}}}$$\mathrm{Input}:\;\Delta w_{in_{j}m}$$=\alpha \underset{v}{\sum }e_{s_{c_{j}^{v}}}dS_{in_{j}m}^{jv}$$\mathrm{for}\;v=1\;\mathrm{to}\;S_{j}$$S_{j}$$\;\mathrm{total}:\;\Delta w_{P_{j^{m}}^{v}}$$=\alpha e_{s_{c_{j}^{v}}}\;dS_{P_{j}^{v}m}^{jv}$
end for
$\mathrm{Output}:\;h=[h_{1,\ldots \ldots ,\;}h_{128}$$],\;h_{i}{}_{0}$
end for

## 6. Performance Evaluation and Discussion 

In this section, we present the LSTM based modified mini-batch discrimination ingestion using the dataset named UNSW-NB15 malware set [20,21].

### 6.1. Metric Analysis

Our study focuses on an LSTM model, comparing Test runs with the following validation testing: accuracy, Model Loss, Receiver Operating characteristic (ROC), and GAN Mode Comparative ROC.

Classification accuracy is determined as a ratio of the number of correct predictions over the total number of input samples using (6)

(6) $$\mathrm{Accuracy}=\frac{\mathrm{Number}\;\mathrm{of}\;\mathrm{Correct}\;\mathrm{Predictions}}{\mathrm{Total}\;\mathrm{Number}\;\mathrm{of}\;\mathrm{Predications}\;\mathrm{Made}}$$

**Figure 5:**
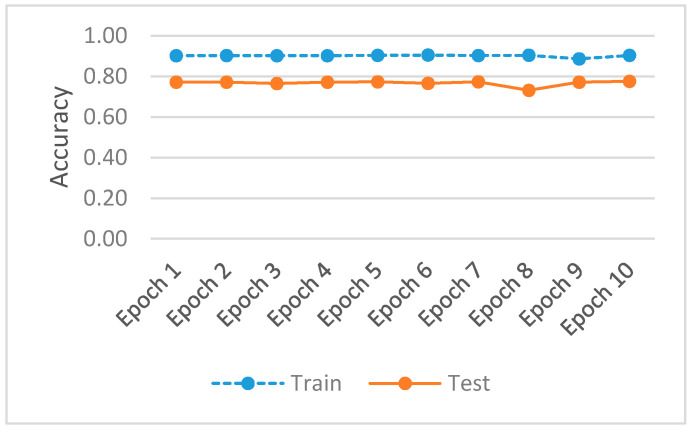
Accuracy metrics for training and testing.

**Figure 6:**
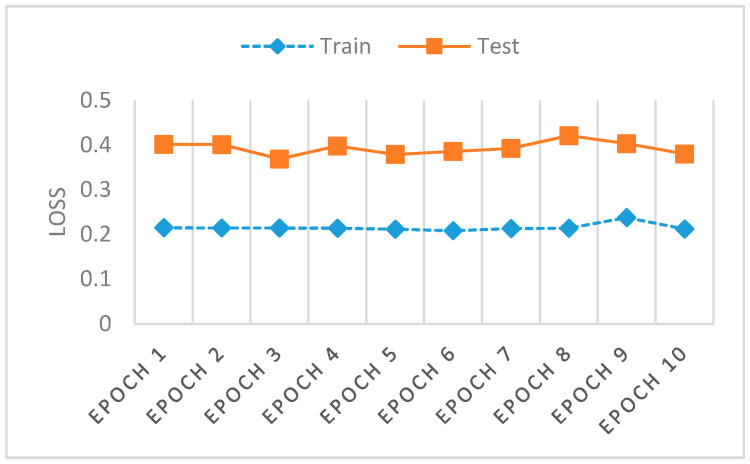
Model Loss metrics for train and test.

Out of 100 epochs, the last 10 epochs are plotted in Figure 5 for accuracy and in Figure 6 for model loss. The layer forecasts an incremental improvement for the GAN model, developed for hazard control flow and botnet anomaly detection by provisioning a deeper architecture. 

Figure 6 shows a model loss for training and testing of our approach with the UNSW NB-15 dataset. Mini-batch options a variation on a gradient descent algorithm, dividing a training dataset by appointed small batches (example batch size 32, 64, 128) used to calculate the model error for recalibration of model coefficients. The current results pace the training and testing within an acceptable margin for the batches allocated from the UNSW NB-15 dataset, offering, as a layered optimizer, prudent assistance to limiting mode collapse. The preferred model loss measures a comparative delta of 0.1545 (0.2142 to 0.3687) and the accuracy delta of 0.1149 (0.887 to 0.7721).

Receiver Operating Characteristic (ROC) curve is plotted with True Positive Rate (TPR), see (7), comparatively to the False Positive Rate (FPR), see (8), where TPR is on the *y*-axis and the *x*-axis is the FPR.

(7) $$\mathrm{TPR}=\frac{\mathrm{TP}}{\mathrm{TP}+\mathrm{FN}}$$

(8) $$\mathrm{FPR}=\frac{\mathrm{FP}}{\mathrm{TN}+\mathrm{FP}}$$

**Figure 7:**
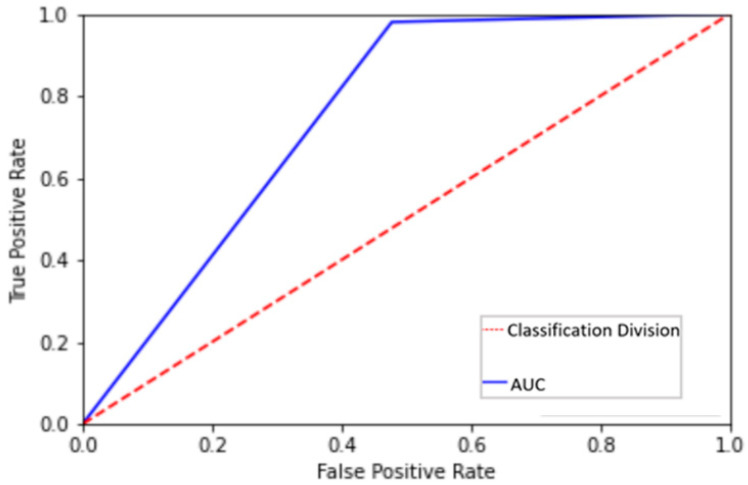
Receiver Operating Characteristics (ROC) Curve.

Changing the number of layers modifies the difficulty of the optimization task, but the interest of the study is to layer improvements in the loss while forecasting an Area Under the Curve (AUC) score beyond 0.5 in the purposed GAN. A higher AUC illustrates the model’s ability to distinguish between classifications. The AUC in Figure 7 is 0.75, which clearly indicates model’s discriminator score of output based on authenticity classification of diversity for our study, as shown in Figure 7.

**Figure 8:**
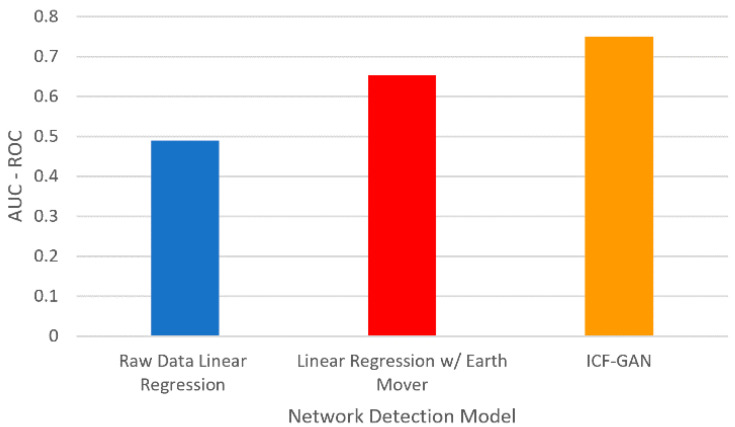
Comparative ROC Scores for different models and the proposed ICF-GAN model.

Next, we plotted the AUC-ROC values in Figure 8 and compared the proposed approach with the approaches such as linear regression with raw data-based and linear regression with earth mover models [50,51]. Our approach ICF-GAN results in the highest AUC-ROC and outperforms the existing method raw data linear regression and linear regression with earth mover [50]. The lower AUC below 0.5 suggests that the model test has limited discriminatory ability, seen in the raw data linear regression model. The linear regression with the earth mover model gives higher AUC than the raw data linear regression model but is lower than our proposed model. The proposed ICF-GAN results in the highest AUC among all three as we consider LSTM based minibatch processing. 

The logistic regression algorithm was impacted by the distribution of the UNSW NB-15 dataset, showcasing improvements after the training [51,52]. ICF-GAN offers a re-search gap enhancement as an increase over that of the augmented logistic regression model. The logistic regression model optimization method is susceptible to overfitting due to the intended batching size, compared to the ICF-GAN outputs, which are ingested within the Control Flow model requiring limited truncation when latent data fooled the discriminator is resampled, yet retaining accuracy. The probability density in the latent sampling, due to the batch size, may limit accurate truncation in comparison to the ICF-GAN model.

In comparison to known PCAP identification models, referencing future mode collapse limiter research, the ICF-GAN model abridges the layered process for flow-based network performance rather than adopting a distance association for data distributional, with a padded encoder structure, for example [51].

Although the reconstruction network learns to map the distribution of noise to all the true data, the enhancement for the generator mapping the entire true data distribution to the noise distribution requires additional training, compared to the presented model, to resolve the model collapse issue holistically in the system control injection model [51].

### 6.2. Comparing Mode Collapse Limiters

As mentioned prior, Wasserstein distance metrics were proposed to provide the measurement of the similarity between the generated distributions and the real data samples. The metric was computed by training a Wasserstein critic on the training set and then evaluating the similarity between the test samples and the generated samples. When the two distributions were similar, the Wasserstein distance was lower. This metric accurately shows mode collapse issues as well as overfitting.

Coverage metrics were used to compute the probability mass of real data covered by the generated samples or identified real samples [18]. For example, the distribution of the generated data and the real data, the kernel density estimation was used to estimate the distribution over real data effective in comparing model performances [18].

Birthday Paradox (BP), a metrics based on the principle used to measure the support size of the learned distribution by a GAN. It states that for *s* support sized *N* distribution, a sample of the square root of the distribution has a high probability of containing a duplicate [18]. The test determines the probability of encountering duplicate pairs in samples [18].

Table 1 shows the comparative results for the proposed ICF-GAN approach and two approaches from the literature RF-NIDS [53] and GAN-FS [54]. We can see that the proposed approach outperforms both RF-NIDS [53] and GAN-FS [54].

Two models are evaluated against the ICF-GAN in part to the model’s intended goal of limiting features of mode collapse, Table 1. Comparing a baseline Random Forest (RF) network-based intrusion detection system (NIDS) model distribution used to evaluate the detection of novel malware, a low Wasserstein distance in the model indicated a decrease in datasets overall feature distributions, respectively, leading to mode collapse [53]. As can be observed, similar between training and testing sets in the NIDS models show similar statistical feature distributions to the totality of malware compared to the ICF-GAN distribution [53].

The Generative Adversarial Networks (GAN) and Feature Selection (GAN-FS) is an oversampling methodology with the perspective of data imbalance elimination [54]. GAN-FS is a hybrid of Gradient Penalty Wasserstein GAN (WGAN-GP), using Analysis of Variance (ANOVA) as a feature-selected rebalancer for low dimensional datasets and RF, scoped in countering training instability [54]. In Table 1, a low Wasserstein distance in the model is indicative of data balancing in the training and test sets with an incremental increase [54].

The coverage and BP scores are similar to the RF-NIDS, with sampling rather than generation. GAN-FS coverage is similar, but improved BP compared to ICF-GAN. The ICF-GAN batch size achieves training stability and controls the accuracy of the estimate of the error gradient during the training while exposing randomness into the data set. During this process, the generator is able to receive more discriminator response information. The chance of overfitting the generator, for our discriminator, decreases, which increases stability and lessens mode collapse.

The higher Wasserstein distance, a metric of probability distributions, in the ICF-GAN model demonstrates better distribution in testing compared to a low Wasserstein distance in the RF-NIDS and GAN-FS models indicating a decrease in the datasets overall feature distributions. The lower coverage by the proposed GAN (0.78) compared to RF-NIDS or GAN-FS provides a lower target data distribution with a probability density that benefits the control flow ingestion process with a lower opportunity of overfitting and higher possibility of inspection in the acknowledgment rule phase beyond STAGE IV. The birthday paradox test, if higher than 50%, is a marker for associates collisions with modal data distribution or GAN model diversity, with the ICF-GAN ranking higher 69.74 than RF-NIDS (64.7) or GAN-FS (48.1) in the study.

## 7. Conclusions

We proposed the ICF-GAN that leverages LSTM with mini-batch discrimination ingestion for intrusion detection systems, which illustrates a better accuracy from Train and Test in the model loss and accuracy for the GAN model developed for hazard control flow and botnet anomaly detection. The ICF-GAP model helps to limit mode collapse with improved accuracy. Our comparative study and numerical results show that the proposed ICF-GAN outperforms the related state-of-the-art approaches.

Our future works include testing the proposed algorithms for other datasets for completeness and use a variety of performance metrics for limiting the mode collapse in GAN models.

## Figures and Tables

**Table 1:** Proposed Approach ICF-GAN Compared To RF-NIDS and GAN-FS Mode Collapse Metrics.

Model	Wasserstein Distance	Coverage	Birthday Paradox
Proposed ICF-GAN	1.83	0.78	69.74
RF-NIDS [53]	0.23	0.83	64.7
GAN-FS [54]	0.18	0.88	48.1

## Data Availability

Datasets analyzed located at https://research.unsw.edu.au/projects/unsw-nb15-dataset (accessed on: 21 April 2021).
